# Ecosystem Engineering by Seagrasses Interacts with Grazing to Shape an Intertidal Landscape

**DOI:** 10.1371/journal.pone.0042060

**Published:** 2012-08-08

**Authors:** Tjisse van der Heide, Johan S. Eklöf, Egbert H. van Nes, Els M. van der Zee, Serena Donadi, Ellen J. Weerman, Han Olff, Britas Klemens Eriksson

**Affiliations:** 1 Community and Conservation Ecology Group, Centre for Ecological and Evolutionary Studies (CEES), University of Groningen, Groningen, The Netherlands; 2 Marine Benthic Ecology and Evolution, Centre for Ecological and Evolutionary Studies (CEES), University of Groningen, Groningen, The Netherlands; 3 Aquatic Ecology and Water Quality Management Group, Department of Environmental Sciences, Wageningen University, Wageningen, The Netherlands; 4 Department of Marine Ecology, NIOZ Royal Netherlands Institute for Sea Research, Den Burg, Texel, The Netherlands; 5 Animal Ecology Group, Centre for Ecological and Evolutionary Studies (CEES), University of Groningen, Groningen, The Netherlands; 6 Department of Biology and Environmental Sciences – Kristineberg, University of Gothenburg, Fiskebäckskil, Sweden; 7 Department of Systems Ecology, Stockholm University, Stockholm, Sweden; Swansea University, United Kingdom

## Abstract

Self-facilitation through ecosystem engineering (i.e., organism modification of the abiotic environment) and consumer-resource interactions are both major determinants of spatial patchiness in ecosystems. However, interactive effects of these two mechanisms on spatial complexity have not been extensively studied. We investigated the mechanisms underlying a spatial mosaic of low-tide exposed hummocks and waterlogged hollows on an intertidal mudflat in the Wadden Sea dominated by the seagrass *Zostera noltii*. A combination of field measurements, an experiment and a spatially explicit model indicated that the mosaic resulted from localized sediment accretion by seagrass followed by selective waterfowl grazing. Hollows were bare in winter, but were rapidly colonized by seagrass during the growth season. Colonized hollows were heavily grazed by brent geese and widgeon in autumn, converting these patches to a bare state again and disrupting sediment accretion by seagrass. In contrast, hummocks were covered by seagrass throughout the year and were rarely grazed, most likely because the waterfowl were not able to employ their preferred but water requiring feeding strategy (‘dabbling’) here. Our study exemplifies that interactions between ecosystem engineering by a foundation species (seagrass) and consumption (waterfowl grazing) can increase spatial complexity at the landscape level.

## Introduction

Spatial heterogeneity is important for the functioning of many different ecosystems, because it can enhance primary productivity, increase the biodiversity and carrying capacity, and stabilize the ecosystem [1]–[4]. Studies from a wide range of terrestrial and marine ecosystems have demonstrated that ecosystem engineers, i.e., organisms that significantly modify their abiotic environment [5], [6], often determine spatial structuring in ecosystems [2]. An important factor often controlling the extent to which the system is modified is the density of the ecosystem engineer [7], while the modified environment in turn also positively or negatively affects the engineer again. In many cases, such feedback mechanisms cause an increase in the spatial patchiness of the ecosystem [8]–[11].

Consumer-resource interactions may also cause patchiness. For instance, plant-herbivore interactions ranging from the arctic tundra to tropical savannahs cause irregular patchy ‘landscape mosaics’ of intensively grazed ‘lawns’ of short vegetation alternating with ungrazed patches of tall vegetation in ecosystems ranging from the arctic tundra to tropical savannahs [3], [12], [13]. Intense grazing of the lawns combined with increased nutrient input by herbivore excrement facilitates growth of consumable and nutrient rich vegetation, while the nutrient-poor, tall vegetation excludes herbivores [12]. Similar to landscapes dominated by ecosystem engineers, these systems are driven by feedbacks. In contrast, however, these feedbacks are not characterized by biotic-abiotic interactions, but mainly driven by trophic interactions.

**Figure 1:**
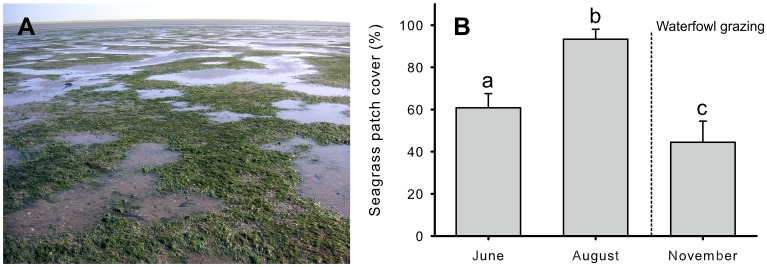
Low-tide exposed hummocks with seagrass alternate with waterlogged, bare hollows in June (A). Seagrass patch cover changed significantly over time (ANOVA: *F*_2,17_ = 66.6, *p*<0.001) from about 61% in June, to over 93% in August, followed by a sudden decrease again to 44% in November due to waterfowl grazing in September and October (B). Error bars indicate SD (number of replicates = 6).

In this study, we investigated the mechanisms behind a spatial mosaic of low-tide exposed hummocks and waterlogged hollows on an intertidal mudflat dominated by the seagrass *Zostera noltii*, which is periodically grazed by waterfowl (Fig. 1A). Using this system as a model, we tested whether an interplay between ecosystem engineering by a foundation species and herbivore grazing activity can lead to patchiness similar to those observed for habitat modification or consumer-resource interactions alone. Intertidal seagrasses like *Z. noltii* are density-dependent ecosystem engineers in the sense that they progressively reduce hydrodynamics and accrete sediment with increasing shoot density [10], [14]–[16]. Grazing by waterfowl is a common phenomenon in seagrass systems. In the Wadden Sea, grazing on *Z. noltii* mainly takes place in autumn by overwintering brent geese (*Branta bernicla*) and widgeon (*Anas penelope*) that migrate from the arctic tundra in Northern Scandinavia and Siberia [17]. The birds use a number of different feeding techniques that depend on the water level. First, upending can be used when the actual water level is still relatively high; next dabbling is employed in areas with a few centimetres of water, and finally grubbing is the most common strategy on completely exposed parts [17], [18]. Although waterfowl can consume significant amounts of both above- and belowground biomass, seagrass is generally not completely removed, but reduced to about 5 to 15% cover. This is because below this threshold feeding becomes energetically unprofitable for the birds, regardless of their feeding mode (‘giving-up density’) [17], [19].

We used a combination of field surveys, a seagrass removal experiment and a spatially explicit model to identify the driving mechanisms behind the observed spatial mosaic. First, we quantified differences in sediment height between hummocks and hollows and measured the patchiness of the system and its change across the season. Second, we performed a seagrass removal experiment to test whether seagrass indeed modified its abiotic environment by sediment accumulation. Third, the effect of grazing by waterfowl on the spatial structure of the system was assessed by bird observations in September and October. Finally, to test whether the identified interactions could indeed explain the observed spatial and temporal patterns, we constructed and analyzed a spatially explicit model based on our field data.

## Field Study Methods

### Site Description

The study was conducted on the intertidal mudflats at Emmapolder, The Netherlands (53° 28′ 0 N, 6° 45′ 0 E) in 2009. It is one of the few areas in the Dutch Wadden Sea where *Zostera noltii* still occurs. Growth of this perennial seagrass starts in late April, peaks in summer, and ends in late autumn (October/November) with seasonal senescence [20].

### Measurements and Experiments

Differences in relative bed level height between hummock and hollows were measured using a Trimble Spectra Precision LL500 Laser Level (Trimble, California, USA). We evaluated the size of seagrass patches at the site by noting the positions of seagrass edges in centimetres along six randomly selected 50 m transects in June, August and November in a 1-hectare study area. Next, seagrass cover was determined by calculating the percentage of mudflat covered by seagrass patches.

To test whether seagrass presence would indeed result in an elevated bed level, we experimentally removed all above- and belowground biomass in six 1 m^2^ plots that were situated on hummocks. Next, we compared the change in bed level from the start in June (a few days after seagrass removal) to the end of the experiment in August to six untreated control plots. In order to remove only seagrass and not the sediment, plywood frames were hammered 20 cm deep along plot edges during low water. Next, the frame was filled with water and all seagrass shoots, roots and rhizomes were removed using a hand rake. After allowing suspended particles to settle, the water was slowly released and the frame gently removed. Height measurements were performed a few days after seagrass removal in June and again in August.

Finally, the effect of grazing by waterfowl was evaluated by bird observation during two low tides (∼4 observation hours per tide) in September and October. For this purpose, we used a Swarovski ATM80-HD spotting scope (zoom ocular 20–60×) placed on a 9 m high dike about 350 m from the site. One observer (JSE) noted the total number of each bird species, recorded the number of feeding birds, their feeding mode and whether they were feeding on hummocks and hollows. To enable an assessment of whether the proportion of feeding birds on hummocks differed from those in hollows, we also noted the percentage cover of hollows and hummocks by visual estimation in a randomly thrown 0.25 m^2^ frame (60 replicates) in the 1-hectare study area.

### Statistical Analyses

Differences in seagrass patch cover between June, August and November, as well as data from the seagrass removal experiment were first tested for normality. As the data were normally distributed, change in patch size was then analyzed using one-way ANOVA. Because Levene’s test showed that variances were not equal, we used a Games-Howell test for post-hoc comparisons. For the seagrass removal experiment, we used a two-tailed independent samples t-test to compare the mean rate of change in sediment height between control and removal plots, and we used a two-tailed paired samples t-test to test for differences in sediment height within treatments before and after the experiment. Bird observation data were analyzed with a Chi-square test.

## Field Study Results

Height measurements in June demonstrated that hummocks were on average 5.8±1.2 cm (mean ± SD; n = 6) higher than hollows. Analysis of transects of seagrass cover in June, August and November demonstrate significant differences between all three periods (ANOVA: F_2,17_ = 66.6, *p*<0.001; Fig. 1B). Seagrass cover was around 61% in June, increased to over 93% in August and decreased again to just over 44% in November.

Experimental seagrass removal in 1-m^2^ plots resulted in a decrease in bed level height, indicating loss of sediment stability. The bed level of the control plots showed a slight increase of 5.8±10 mm (mean ± SD), but this was not significant (paired t-test: t_5_ = 1.41, *p* = 0.218). The bed level height in the experimental removal plots decreased significantly compared to the control plots (t-test: t_10_ = 4.61, *p*<0.001) and the removal plots also decreased significantly in height over time (paired t-test: t_5_ =  −5.86, *p* = 0.002) by 17.6±7.3 mm (mean ± SD).

**Figure 2:**
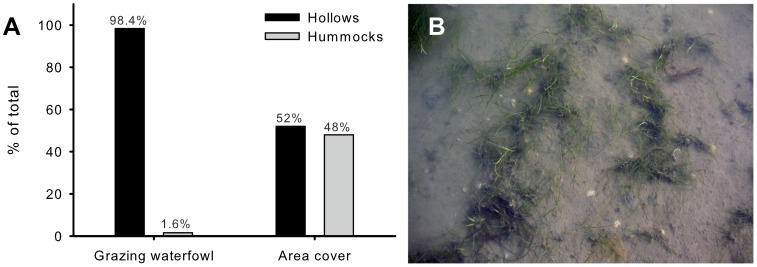
Over 98% of all grazing waterfowl was feeding in hollows (A), whereas hollows covered only 52% of the entire 1-hectare area (χ^2^ = 106.89, df = 1, p<0.001). Visual observations showed obvious grazing scars in the hollows (B): trampling and beak marks, uprooted plants, floating leaves, and a removal of seagrass up to 90% of the original biomass. Hummocks, in contrast, were minimally impacted.

Bird observations showed that mixed flocks of around 150 brent geese (70%) and widgeon (30%) grazed at the site in September and October, resulting in densities of around 200 individuals per hectare. Even though hollows covered only 52% of the area, over 98% of the feeding birds (80% of all) fed in hollows (χ^2^ = 106.89, df = 1, *p*<0.001; Fig. 2A). Visual inspection revealed that birds mostly fed by dabbling in hollows and by grubbing on hummocks. Furthermore, hollows showed distinct signs of waterfowl grazing (e.g., trampling and beak marks, uprooted plants, floating leaves) and an eventual reduction of about 90% of the original seagrass cover (Fig. 2B). In contrast, hummocks were hardly impacted.

## Model Description

Results from the field study suggest that the observed two-state mosaic of hummocks and hollows resulted from an interaction between density-dependent habitat modification by seagrass and selective grazing by waterfowl. Dense seagrass stands accrete sediment, which causes elevation of the bed level compared to the bare surroundings. However, sediment accretion is disrupted in patches that are grazed by waterfowl in autumn. This results in low-tide waterlogged hollows in the meadow that are colonized by seagrass during the growth season, but are then selectively grazed again by the waterfowl – most likely because their preferred feeding strategy (dabbling) is only possible in the water-logged hollows [18].

To test whether these interactions could indeed explain the observed patchy landscape, we constructed a minimal, spatially explicit computer model based on our empirical data (see table 1 for data sources). The model describes changes in seagrass shoot density and bed level height in two differential equations:


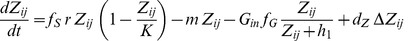
(1)


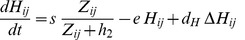
(2)

*Z_ij_* and *H_ij_* are the seagrass shoot density (shoots m^−2^) and bed level height (m) in grid cell i, j respectively. The Laplace gradient operators *d_Z_* and *d_H_* (day^−1^) describe diffusion of seagrass and sediment to neighboring grid cells to mimic lateral dispersion of seagrass and to prevent unrealistically large differences in bed level height between bordering cells (i.e., very steep slopes). Function *f_S_* (*t*)_,_ with *t* as time, describes the seasonal differences in seagrass growth:


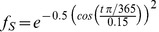
(3)

The maximum net logistic growth rate is described by *r* (day^−1^), *m* is the mortality rate of seagrass due to natural senescence (day^−1^) and *K* is the carrying capacity (shoots m^−2^). The maximum sedimentation rate of suspended sediment is described by *s* (m day^−1^), maximum erosion rate *e* (day^−1^) is calculated as the sedimentation rate *s* divided by the maximum bed level height *H_max_* (m) and *h_2_* (shoots m^−2^) is the seagrass density at which the sedimentation rate is 50% of the maximum sedimentation rate *s*. *G_in_* is the maximum feeding rate for individual birds (sh ind^−1^ day^−1^) and *h_1_* (shoots m^−2^) is the half saturation constant for the density dependent feeding rate on seagrass. Finally, function *f_G_* describes the local number of birds per m^2^ which is dependent on the season, seagrass density and the bed level height. Waterfowl are absent during most of the season (*f_G_* = 0), but are present on a gridcell in autumn between days 243 and 334 of the year (*t_y_*) provided that local seagrass density is above the giving-up density threshold (*GUD*; shoots m^−2^):


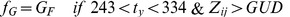
(4)

The number of feeding birds (*G_F_*, ind m^−2^) depends on the number of present waterfowl and the elevation of the grid cell:


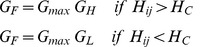
(5)

Where *G_max_* describes the maximum number of birds (ind m^−2^), *G_H_* and *G_L_* are the feeding fractions on high and low parts respectively and *H_c_* is the critical elevation threshold (m).

**Table 1:** Default parameter settings of the spatially explicit model.

	Default	Unit	Description	Source
**Variables**				
*Z*		sh m^−2^	Seagrass shoot density	
*H*		m	Bed height	
*G_F_*		ind m^−2^	Number of feeding waterfowl	
**Parameters**				
*r*	0.4	day^−1^	Relative growth rate	*
*m*	0.007	day^−1^	Mortality rate	*
*K*	5976	sh m^−2^	Carrying capacity	*
*s*	0.00035	m day^−1^	Sedimentation rate	*
*H_max_*	0.2	m	Maximum bed height	*
*e*	*s/H_max_*	day^−1^	Erosion rate	
*h_1_*	594	sh m^−2^	Half rate constant for seagrass feeding rate	1,*
*h_2_*	1500	sh m^−2^	Half rate constant for sedimentation rate	*
*G_max_*	0.03	ind m^−2^	Maximum number of waterfowl	*
*G_in_*	16967	sh ind^−1^ day^−1^	Maximum intake rate for waterfowl foraging	1,*
*GUD*	0.1 *K*	sh m^−2^	Giving-up density for waterfowl foraging	1,2
*G_L_*	0.984		Fraction of waterfowl feeding in hollows	*,2
*G_H_*	0.016		Fraction of waterfowl feeding on hummocks	*
*H_C_*	0.08	m	Critical bed height for waterfowl foraging	*
*d_Z_*	0.2 *r*	day^−1^	Diffusion rate constant for seagrass	±
*d_H_*	0.2 *e*	day^−1^	Diffusion rate constant for bed height	±

(*) Obtained from field data, (1) Percival & Evans (1997), (2) Ganter (2000), (±) Gradient operators *d_Z_* and *d_H_* were set at 20% of the maximum growth and erosion rate respectively. Parameters measured in the field resulted either from fieldwork published in this study or from [10].

To investigate whether the model could indeed generate the spatial and temporal patchiness observed in the field, we randomized seagrass density and sediment height between zero and half of their maxima (0.5 *K* and 0.5 *E_max_* respectively) across a 200×200 grid and ran the model at default parameter settings (Table 1) for 100 years. Additionally, we performed a bifurcation analysis on the maximum number of waterfowl and the maximum sedimentation rate in a non-spatial version of the model (i.e., a ‘single grid cell’ system). Bistability in this non-spatial model is a necessary condition for mosaics in the spatial version of the model; i.e., alternative ‘hollow’ and ‘hummock’ states need to be possible at the same external conditions. To examine in what ranges of sedimentation rates and waterfowl densities such alternative stable states are possible, each parameter was gradually increased in small steps. After each increase, the model was allowed to stabilize and the average shoot density and sediment height across the year were recorded. Next, the same procedure was also performed in a backward manner, i.e., a gradual decrease of parameter values. We used the results from this bifurcation analysis to construct a two-dimensional plot of waterfowl numbers (*G_max_*) and sedimentation rate (*s*). The range of parameters where alternative stable states are present in this model indicates the parameter space where mosaics are possible in the spatial model. Note that, although local bistability is a requisite for mosaics, patch size and distribution in the spatial model will also depend on the initial conditions of *Z* and *E*.

## Model Results

**Figure 3:**
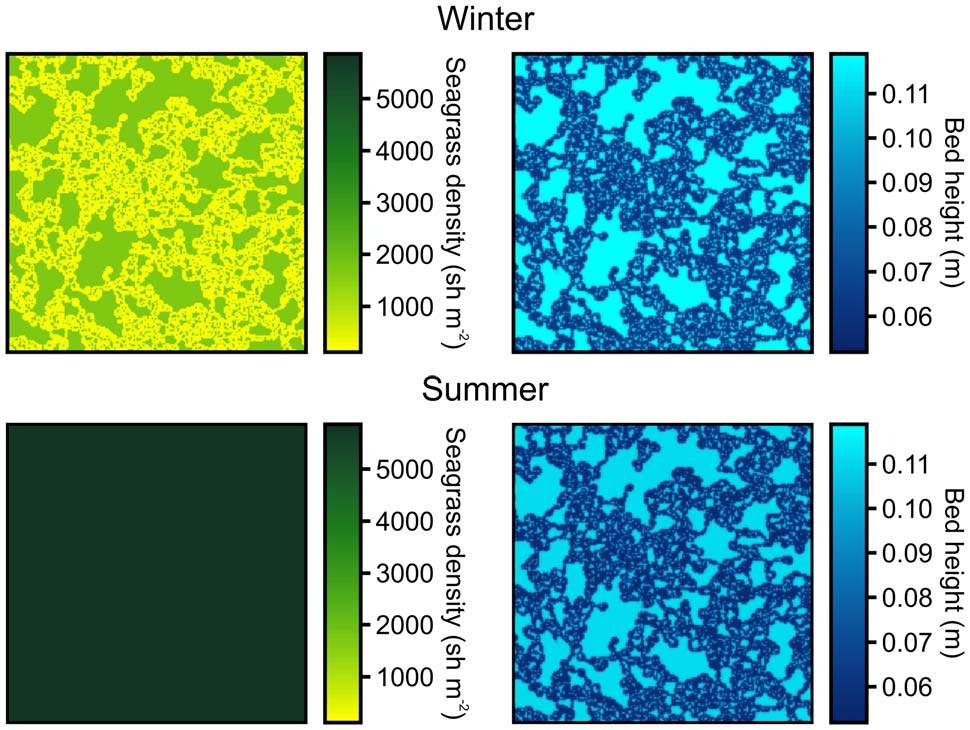
Model simulations at defaults settings demonstrated that interactions between sediment accretion by seagrass and selective grazing of low parts by waterfowl in autumn could indeed explain the observed temporal and spatial patterns in the field.

Model simulations confirmed that the identified interactions between sediment accretion by seagrass and grazing by waterfowl could indeed explain the observed spatial and temporal patterns (Fig. 3, Movies S1 & S2). Similar to the field situation (Fig. 1B), seagrasses covered hummocks throughout the year. Hollows were colonized during the growth season, leading to full cover in summer. However, these depressions are grazed in autumn, thereby preventing these patches to accumulate sediment and returning the system to its initial patchy state.

**Figure 4:**
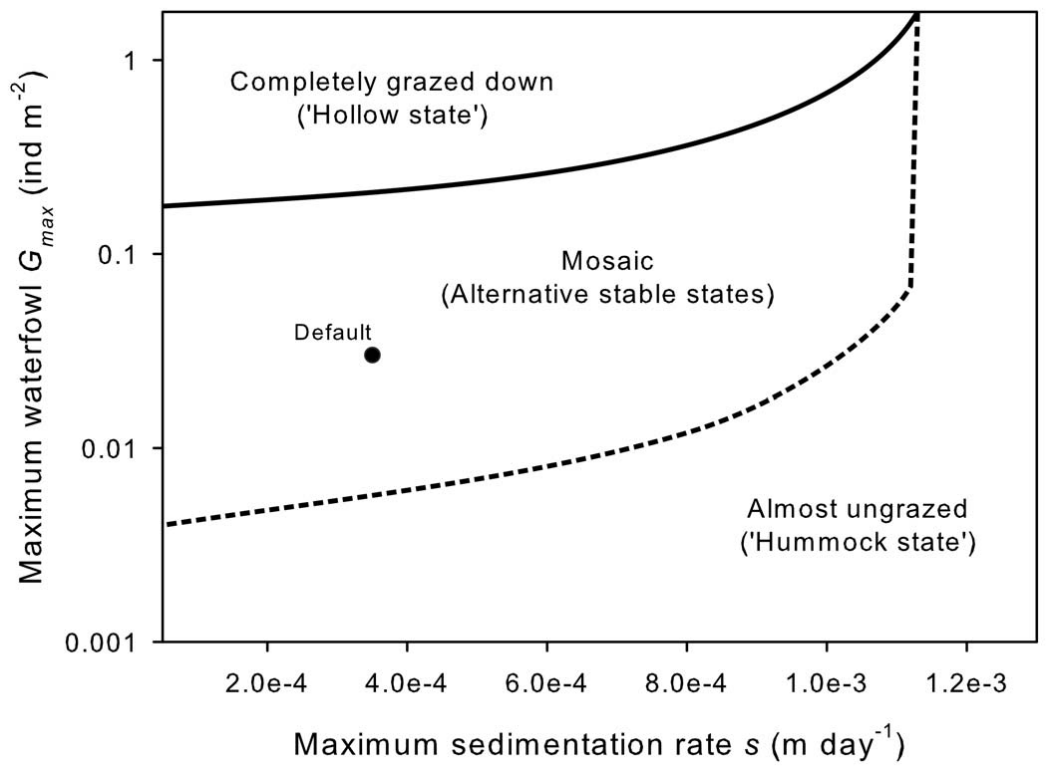
A two-dimensional bifurcation analyses of the maximum sedimentation rate *s* and the maximum number of waterfowl *G_max_* (on a log-scale) in the non-spatial version of the model demonstrates that landscape mosaics can appear over a wide range of parameter settings. The solid line indicates the threshold between a mosaic state and the conditions where seagrass is completely grazed down in autumn. A state with an elevated bed level and low grazing is the only possible state below and right of the dashed line. Default parameter settings are indicated by the black dot. The effect of sedimentation rate was analysed in the range of 0.05 to 1.13 mm day^−1^, bird densities were tested in the range of 0.001 to 2 ind m^−2^. Note that formation of mosaics is not possible in the absence of sedimentation and/or birds.

The two-dimensional bifurcation analysis of the maximum sedimentation rate (*s*) and the maximum number of waterfowl (*G_max_*) in the non-spatial model demonstrated the possibility for spatial mosaics over a wide range of parameter settings (Fig. 4). At low sedimentation rates, bistability appears just above zero waterfowl numbers until well over the observed number of waterfowl in the field. At extremely high waterfowl numbers, the mosaic collapses to a state where the complete meadow is grazed in autumn, but is still able to recover in the next growth season. When the sedimentation rate is set extremely high, hollows can accumulate sufficient sediment in one growth season the reach a ‘hummock state’, thereby excluding most of the waterfowl in the meadow.

## Discussion

Spatial heterogeneity in ecosystems can be caused by abiotic variability, but can also result from feedback mechanisms [8], [21]. Such feedbacks emerge when organisms significantly modify their environment (i.e., ecosystem engineering) [2], [21], but can also be caused by plant-herbivore, predator-prey and host-parasitoid interactions [12], [13], [22], [23]. In this study, we report on a spatial mosaic of low-tide exposed hummocks and waterlogged hollows that results from an interaction between sediment accretion by seagrass and selective grazing by waterfowl, thereby illustrating that interactions between ecosystem engineering by a foundation species and grazing can cause spatial structuring in ecosystems. This finding is important because studies ranging from arctic to tropical environments and from terrestrial to marine ecosystems have demonstrated that spatial heterogeneity is often essential for ecosystem functioning [1]–[4].

Our study exemplifies how an interplay between biotic and abiotic factors can spatially structure intertidal seagrasses. The possibility of patchiness being solely driven by underlying abiotic heterogeneity or seagrass ecosystem engineering can be discarded because seagrasses colonized bare areas in summer and were observed to only retreat again due to waterfowl grazing. Similarly, our observations also reveal that waterfowl grazing alone is not sufficient to explain the observed spatial patchiness and temporal trends, as observations showed that grazing intensity was strongly driven by sediment height, which in turn was dependent on sediment accretion by seagrass. Although our study captured the most important structuring mechanisms, some processes have been disregarded or were simplified in both the model and the field experiments. Examples of factors possibly affecting the observed patchiness are bioturbation by infauna [10], local differences in current velocity and sedimentation, and stochastic events like storms or desiccation of seagrasses at low tide during days with high temperatures [24]. Another simplification is our description of grazing in the model. Here, grazing is described as a process that only removes seagrasses from the system. In reality, depending on the feeding mode (i.e., dabbling, grubbing), grazing by waterfowl also decreases the cohesiveness of the sediment, thereby increasing erosion in the impacted areas. As dabbling (the preferred feeding strategy) directly results in sediment resuspension in the hollows, this will most likely have the most pronounced effect on sediment erosion. This suggests that we may have underestimated the overall effect of waterfowl grazing on erosion of grazed patches in our model, which would in turn imply that the ‘hollow-state’ is in reality more resilient than in the default setting of our model. Nevertheless, our bifurcation analysis (Fig. 4) clearly demonstrates that alternative stable states exist over a much wider range of grazing intensities and sedimentation-erosion balances than those measured in the field, indicating that an under- or overestimation of grazing and/or erosion does not fundamentally alter our results.

The spatial structure of seagrass ecosystems is often attributed to abiotic factors such as wave action, currents, sediment transport and light [25]–[29]. Recent studies, however, have shown that seagrasses are strong ecosystem engineers that often improve their own conditions, for instance by lowering nutrient levels, attenuating hydrodynamics and accumulating sediments [15], [30], [31]. Moreover, when such positive feedbacks interact with negative feedbacks, it may lead to spatial self-organization in seagrasses [24]. Furthermore, intensive grazing by waterfowl, turtles, dugongs, manatees and urchins has been demonstrated to have significant effects on the spatiotemporal structure and overall productivity of seagrasses as well [17], [32]–[34]. Over the last century, seagrass meadows have been increasingly disturbed by human activities (e.g., eutrophication, siltation, dredging), resulting in dramatic and large-scale losses worldwide that were in many cases unexpected [15], [35], [36]. Our results support the notion that consideration of biological interactions between seagrasses and associated organisms may be crucial for conservation and restoration efforts in many seagrass ecosystems [37], [38].

Spatial patchiness caused by ecosystem engineering interacts with both abiotic stress and grazing in various ecosystems [21], [24], [39]–[41]. However, grazing in these previously studied systems is not part of the structuring feedback mechanisms, and the disruption of these feedbacks by grazing therefore typically induces loss of spatial structure [9], [39], [40]. In contrast, the interaction with grazing is the actual cause of spatial patchiness in our system. Furthermore, in contrast to results from resource-limited systems, our model does not predict a complete collapse of the vegetation above a certain threshold for grazing [39], but rather a homogeneous state of intense periodic grazing (Fig. 4). The seagrass meadow in our model does not collapse because (1) seagrass growth is not resource-limited and (2) waterfowl grazing is periodic and does not continue below 10% of the maximum seagrass density (‘giving-up density’). These results are in agreement with other studies on waterfowl grazing in intertidal seagrasses that show that seagrass survival and production are either not markedly impacted [17] or even facilitated by waterfowl grazing [33], [42]. Our study therefore supports the notion that the driving mechanisms behind spatial structuring should be well understood before using patchiness as an indicator of stress in ecosystems [24], [41].

## Supporting Information

Movie S1 **A 10-year simulation of seagrass growth dynamics on a 100×100 cell grid.**
(MP4)

Movie S2 **A 10-year simulation of sediment accumulation and erosion dynamics on a 100×100 cell grid.**
(MP4)
